# Summer Complaints

**Published:** 1878-04

**Authors:** 


### SUMMER COMPLAINT.

How many a sweet child is torn from the arms of its parents, in a great city like this, every day during summer, the "Weekly Reports" fully testify. And what hopes are blasted thereby; what wounds are made, never to be wholly healed on earth; what houses are desolated; what hearts broken; never can be known. But we know, that a large part of all this is avoidable. To those who cannot leave the city, we recommend to keep their children in the second or third stories of their dwellings, until after breakfast, and to confine them to the same from sundown until the next morning.

